# The effect of vitamin C on procalcitonin biomarker in community‐acquired pneumonia

**DOI:** 10.1186/s12948-021-00140-1

**Published:** 2021-03-03

**Authors:** Mahsa Nikzad, Hamid Reza Banafshe, Mansooreh Momen-Heravi, Hamed Haddad Kashani, Maryam Shiehmorteza

**Affiliations:** 1 Department of Clinical Pharmacy, Faculty of Pharmacy, Pharmaceutical Sciences Branch, Islamic Azad University, Tehran, Iran; 2 Department of Pharmacology, School of Medicine, Kashan University of Medical Sciences, Kashan, Iran; 3 Infectious Disease Research center, Kashan University of Medical Sciences, Kashan, Iran; 4 Anatomical Sciences Research Center, Institute for Basic Sciences, Kashan University of Medical Sciences, Kashan, Iran

**Keywords:** Interlukin6, C-reactive protein, Procalcitonin, Community acquired pneumonia, Vitamin C

## Abstract

**Introduction:**

Community acquired pneumonia (CAP) is a prevalent low respiratory infection. Diagnosis is based on clinical symptoms, radiologic evidence and culture. Biomarkers such as IL6, CRP and procalcitonin are helpful in diagnosis. Procalcitonin is a soluble biomarker in serum that increase in systemic inflammation and bacterial infections. People with normal procalcitonin have low risk to infect pneumonia. Patient with CAP have more oxidative stress than normal people. Studies show that receiving vitamin C can reduce incidence of pneumonia. The present study was designed to evaluate the effect of vitamin C supplement on procalcitonin biomarker in patient with CAP.

**Methods:**

Patients with CAP who passed inclusion and exclusion criteria after obtaining informed consent, were assigned randomly in two groups of drug and placebo. The drug group received vitamin C (1000 mg/d) daily and medications that physician prescribed for treating CAP for 10 days and placebo group received placebo and medications that physician prescribed. The serum level of procalcitonin was measured at the beginning of the study and after 10 days of intervention.

**Results:**

35 patients finished the study. Serum level of procalcitonin on the first and tenth day did not show any significant difference between drug and placebo groups.

**Conclusions:**

To clarify the relationship between the effects of vitamin C on procalcitonin in CAP, a larger sample size is required.

## Introduction

Community-acquired pneumonia (CAP) is defined as pneumonia that accurses as a result of presence in the health care system (such as hospital, long-term treatment and chronic uses of antibiotics [1]. CAP is one of the biggest causes of Infection-caused deaths in developed countries [2]. The prevalence causes of CAP are *Streptococcus pneumonia, Haemophilus influenza, and Staphylococcus aureus* [3]. CAP is diagnosed by clinical symptoms like cough, fever, Shortness of breath and chest radiography. The type of pathogen diagnosis is determined by microbial culture [4]. The new method for infection diagnosis and treatment responses is using of biomarkers [5]. These biomarkers are including C-reactive proteins (CRP), Lactate, interleukin 6, interleukin 8 and procalcitonin [6]. CRP sensitivity for sepsis diagnosis is not significant and its plasma peak can’t show the infection severity and inflammation. Also it can increase in some trauma or other diseases [7]. Based on the reports, Procalcitonin (PCT) is the most accurate laboratory test for bacterial infection with 89% sensitivity and 94% specificity [8]. PCT as a diagnostic factor can significantly reduce the antibiotic prescription and the treatment duration in lower respiratory infection patients [9, 10]. In CAP the significant increasing of oxidative stress reactions and inflammatory factors production occurs in peripheral vessels [11]. Antioxidants reduce tissue oxidative damage and rapid inflammatory responses by affecting their activation genes [12]. Vitamin C is an antioxidant that significantly reduces the respirational symptoms in most patients [13]. The evidence shows that ascorbic acid can probably have the anti-viral activity in vivo [14]. It is currently believed that people with low vitamin C levels are disposed to infection and oxidative stress [15]. According to the high cost of treatments with antibiotics and the harmful effects of increased number of patients and their treatment duration, using new methods for reduction of these items is necessary. Considering the effect of serum procalcitonin levels as a recovery indicator on antibiotic therapy duration and also the effect of vitamin C as an antioxidant on the recovery of pneumonia patients, the aim of this study was the determination of the exact relation between the effects of this supplement on PCT level in vitamin C and placebo receivers.

## Material and method

At first, 40 CAP patients were included in this study and divided into two groups. Finally, 35 patients, 17 in vitamin C (1000 mg/d) receiver group and 18 in placebo receiver group, completed the study. 10 numbers of Vitamin C and placebo tablets were taken by patients in 10 days. On the first and tenth day, 2 CC of venous blood sample were taken from patients to measure the serum level of procalcitonin. Serum PCT level was evaluated by its specific kit using enzyme*-*linked fluorescence assay (ELFA) method.

### Statistical analysis

Data were analysed by SPSS version 17.0 software. Statistical tests such as T-test, Chi-square and Fisher exact were used. Also, the Kolmogorov-Smirnov test was used for assessing data distribution. The PCT serum level data were not normally distributed, so mann-whitney u and Wilcoxon signed-rank test were used to examine the changes in the level of this factor. T-test and Paired-T tests were used for data that had normal distribution such as CRP, Erythrocyte sedimentation rate (ESR) and white blood cell (WBC) data. P-Value < 0.05 was considered significant.

### Ethical consideration

**Fig. 1:**
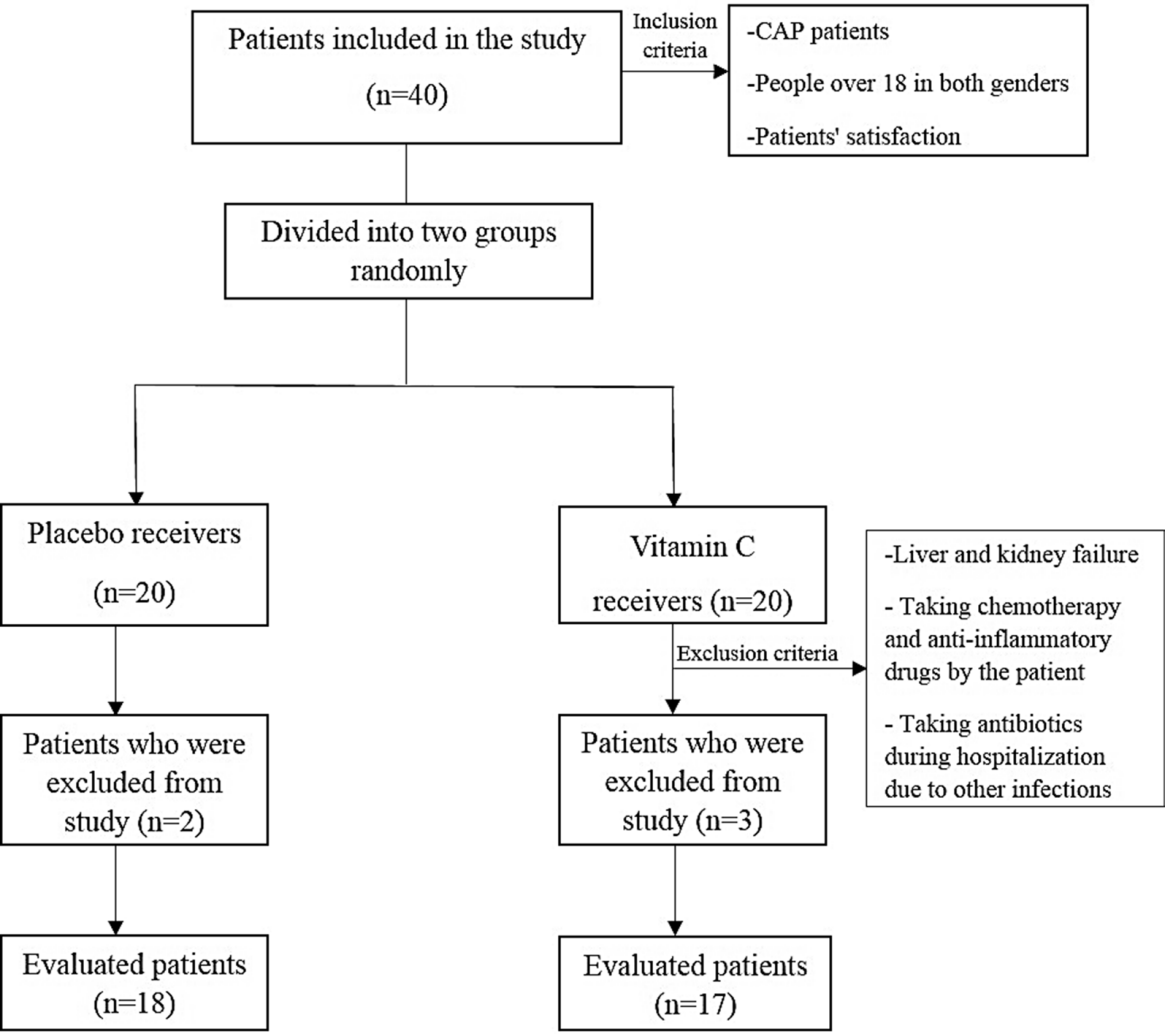
Flowchart of patients who entered the study

 The study was approved by the ethics committee of the Pharmacy Faculty, Islamic Azad University, Tehran medical branch with ID IR.IAU.PS.REC.1396.220. The Patients were informed about the research and all patients signed the consent form and ethical principles were observed according to the requirements of the ethics committee (Fig. 1).

## Results

PCT, CRP, ESR and WBC serum level were evaluated on the first and tenth day of the study in both groups. PCT serum level had no significant differences on first and tenth day. In both groups, the serum level of this biomarker decreased but these changes were not significant. However, Wilcoxon signed-rank test showed that ten-day-treatment for pneumonia with vitamin C or placebo made a significant change in procalcitonin serum level (Table 1).

**Table 1:** Wilcoxon signed-rank test showing the PCT level variation

	Vitamin C receiver		Placebo receiver	
PCT	First day	Tenth day	First day	Tenth day
Average	0.8303	0.7947	0.0772	0.043
Standard deviation	2.275	2.267	0.07805	0.0573
	Z=−3.214 Sig = 0.001		Z=−3.386 Sig = 0.001	

There was no significant change in the ESR levels in both groups on the first and last day. In placebo receivers, unlike the vitamin C receivers, there was an increase in ESR level during the hospitalization. Paired-T test showed that pneumonia treatment in addition to vitamin C or placebo made no significant difference in ESR level (Table 2).

**Table 2:** Paired-T test showing the ESR level variation

	Vitamin C receiver		Placebo receiver	
ESR	First day	Tenth day	First day	Tenth day
Average	38.7647	33.800	35.6250	37.2143
Standard deviation	23.0828	27.7699	20.8130	24.7018
	T=-0.92 Sig = 0.37		T=-0.447 Sig = 0.662	

CRP level on the first and last day of hospitalization didn’t significantly change and this biomarker decreased in both groups. According to the Paired-T test, treatment with prescription drugs in addition to vitamin C or placebo made a significant decrease in CRP level in both groups. But there was no significant relationship between the differences in the two groups (Table 3).

**Table 3:** Paired-T test showing the ESR level variation

	Vitamin C receiver		Placebo receiver	
CRP	First day	Tenth day	First day	Tenth day
Average	53.933	29.1429	46.4312	24.8571
Standard deviation	29.2708	26.7491	31.9452	19.2181
	T=−2.873 Sig = 0.014		T=−3.046 Sig = 0.009	

According to the p-values, WBC levels had no significant differences in two groups during the hospitalization and there was a decrease in two groups. Paired-T test showed that pneumonia treatment in addition to vitamin C made a significant decrease in WBC level but in placebo receivers, it didn’t significantly decrease (Tables 4,  5).

**Table 4:** Paired-T test showing the WBC level variation

	Vitamin C receiver		Placebo receiver	
WBC	First day	Tenth day	First day	Tenth day
Average	8.3341	7.0867	7.7833	7.1743
Standard deviation	2.8563	2.3079	2.8824	2.7765
	T=−2.180 Sig = 0.047		T=−2.047 Sig = 0.061

**Table 5:** The average of PCT, CRP, ESR, and WBC levels on the first and tenth day

Variables		Average	Standard deviation	P-value
PCT (first day)	Vitamin C receiver	0.8303	2.27549	0.93
	Placebo receiver	0.0772	0.07805	
PCT (tenth day)	Vitamin C receiver	0.7947	2.26754	0.98
	Placebo receiver	0.0430	0.05738	
ESR (first day)	Vitamin C receiver	38.7647	23.08281	0.685
	Placebo receiver	35.6250	20.81306	
ESR )tenth day)	Vitamin C receiver	33.800	27.7699	0.730
	Placebo receiver	37.2143	24.70185	
CRP (first day)	Vitamin C receiver	53.933	29.2708	0.5
	Placebo receiver	46.4312	31.9452	
CRP (tenth day)	Vitamin C receiver	29.1429	26.7491	0.63
	Placebo receiver	24.8571	19.2181	
WBC (first day)	Vitamin C receiver	8.3341	2.85633	0.57
	Placebo receiver	7.7833	2.88245	
WBC (tenth day)	Vitamin C receiver	7.0867	2.30799	0.92
	Placebo receiver	7.1743	2.77657	

## Discussion

In the current study, PCT serum level was evaluated on the first and tenth day. This biomarker was significantly decreased in both groups but this decrease was not significant in vitamin C and placebo receivers. Therefore, vitamin C didn’t affect the PCT level. Boussekey in his study assessed the value of PCT for CAP diagnosis and showed that PCT > 2ng/ml had a relation with increased incidence of blood bacteria, sepsis shock, organs failure and death [16]. In Christ-Crain’s study, it was shown that the use of PCT as a treatment guide decreased the antibiotic exposure and treatment duration compared with standard group [17]. Numerous studies show that low levels of vitamin C in plasma, white blood cells, and urine occurs during various infectious diseases, which is not only due to inadequate diet of this vitamin but also occurs under the influence of infection physiological changes. Alpha a Fowler et al. had a study on sepsis patients in 2014. The patients were divided into two groups: ascorbic acid receivers and placebo receivers (5% dextrose serum). PCT and CRP serum levels were evaluated in both groups. No adverse events were observed in ascorbic acid receivers. CRP level was significantly reduced in ascorbic acid receivers compared to the initial value and placebo group. Also, PCT level in placebo receivers increased in the first 24 h and it was significantly reduced in vitamin C receivers compared to its initial value in the first 48 hours. This study showed that ascorbic acid receiving can lead to a rapid decrease of inflammatory biomarkers in severe sepsis [18]. Another inflammatory biomarker is CRP. In the current study CRP, ESR, and WBC levels were evaluated on the first and tenth day. Changes of these biomarkers during the hospitalization had no significant relationship between the two groups. A study in 2007 indicated that daily evaluation of CRP can be useful for diagnosis of CAP patients. This biomarker had a better anticipating of patient status compared with other common markers like body temperature and leukocyte level evaluation. This study proved that rapid CRP decrease in patients led to a shorter duration of antibiotic therapy with the same effect and less toxicity. Therefore CRP measurement in emergency cases led to the treatment costs decrease [19].

## Conclusions

According to the Wilcoxon signed-rank test, ten-day-treatment with vitamin C or placebo made a significant change in PCT serum level in both groups but this change between two groups was not significant. For more certainty, further studies in a larger community are needed.

## Data Availability

The dataset used in this study is available with the authors and can be made available upon request.

## Abbreviations

CAP: Community acquired pneumonia
PCT: Procalcitonin
WBC: white blood cell
ESR: Erythrocyte sedimentation rate
CRP: C-reactive protein
